# Supplementary material for: A global transcriptomic analysis of Staphylococcus aureus biofilm formation across diverse clonal lineages
Source: Microb Genom. 2021 Jul 6;7(7):000598. doi: 10.1099/mgen.0.000598 (PMC8477394; doi:10.1099/mgen.0.000598)

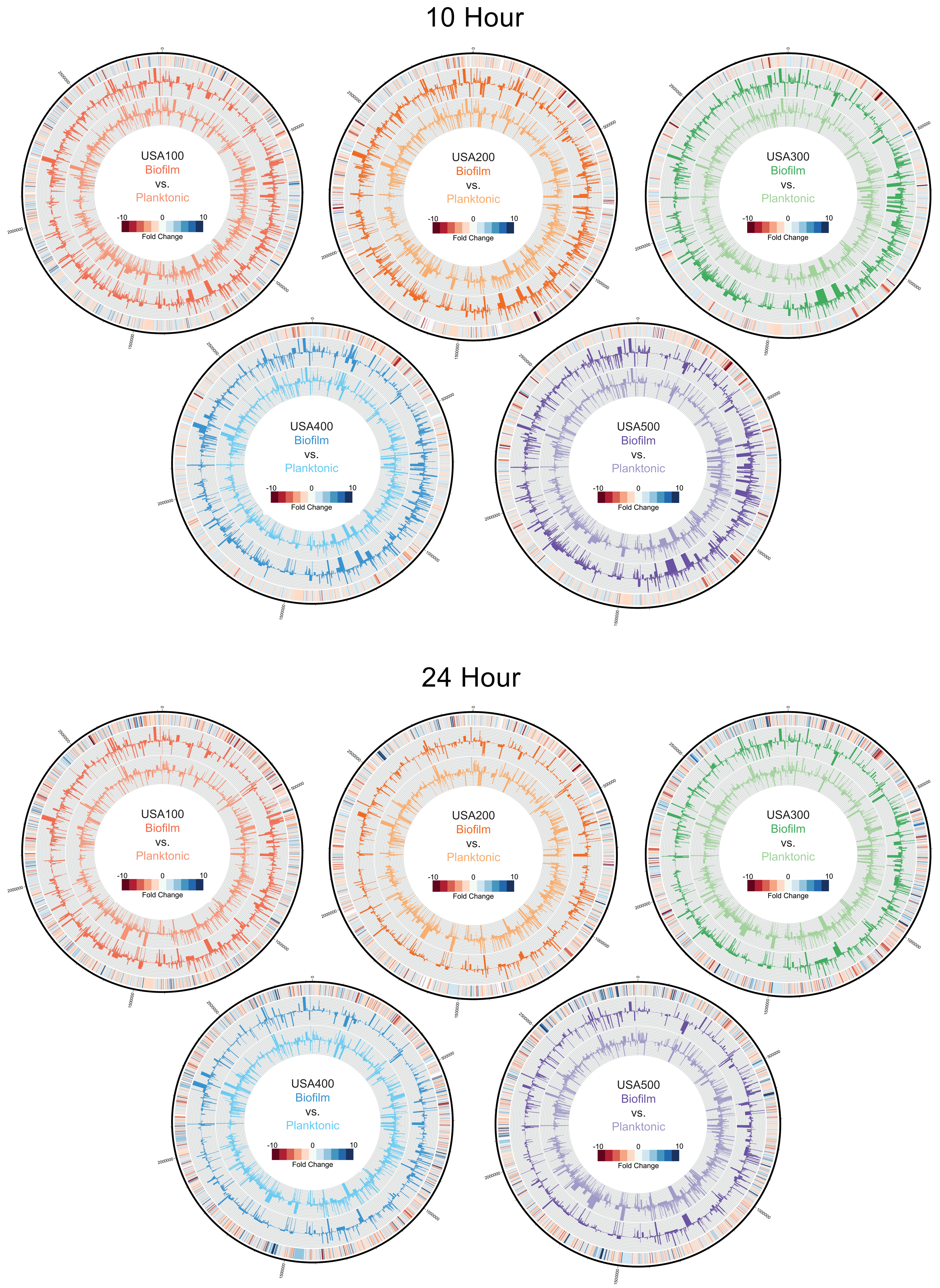

Figure S1: *S. aureus* biofilms exhibit differential expression compared to planktonic cell populations. Genomic maps were created for each strain depicting changes in the planktonic (inner histograms, light colors) and biofilm (outer histograms, dark colors) transcriptomes at 10 h (top) and 24 h (bottom) reported as TPM expression values. The outermost circle is a heat map demonstrating fold change in expression, where red or blue indicates higher expression in the biofilm or planktonic cell population, respectively.

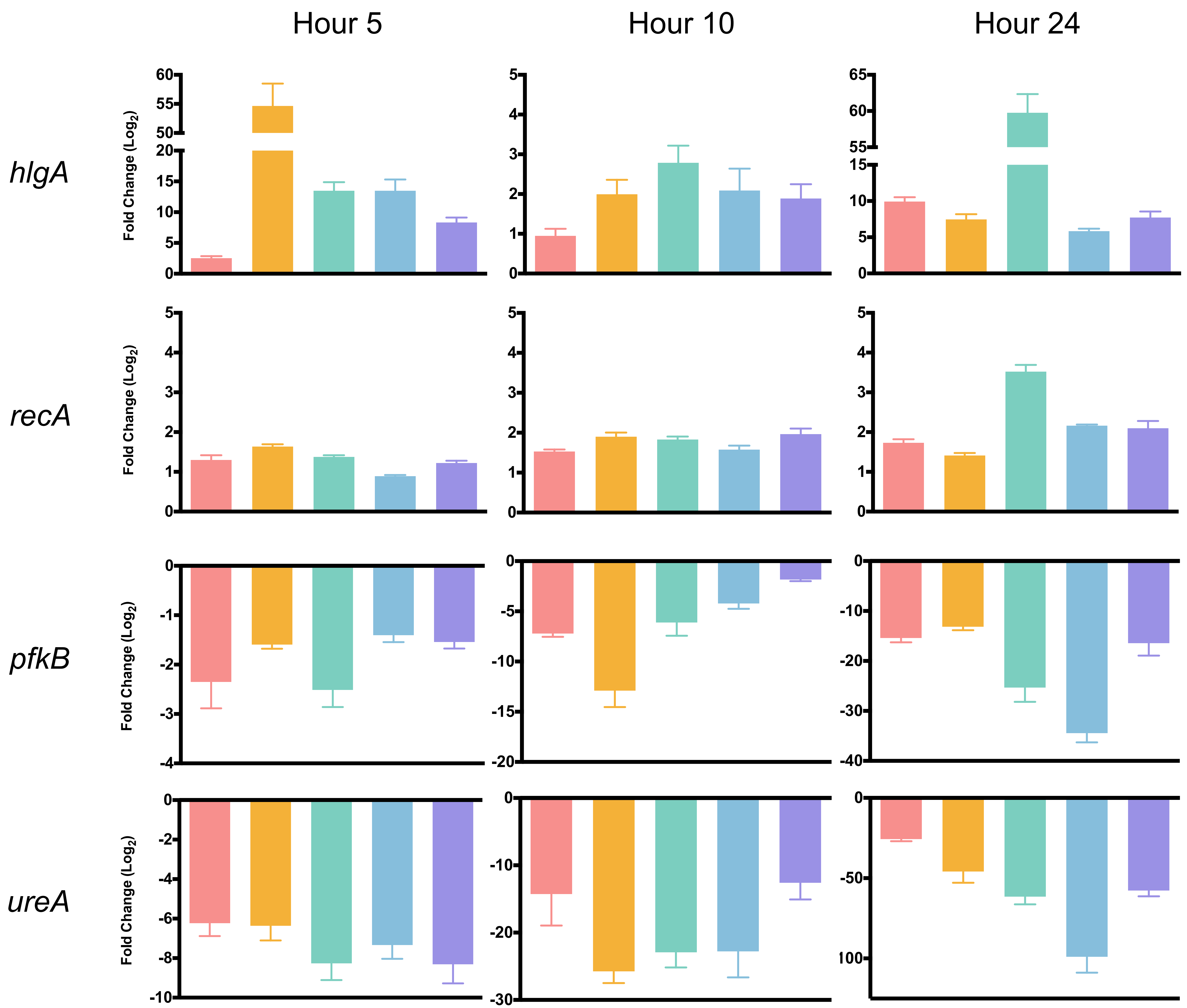

Figure S2: RT-qPCR validation of RNA-seq findings. RT-qPCR was performed using gene specific primers for four randomly selected genes (left), each timepoint (top), and each strain (USA100, red; USA200, orange; USA300, green; USA400, blue; USA500, purple). Expression levels were normalized to 16s rRNA and calculated using the $2^{-\Delta\Delta Ct}$ method. Data is reported as mean fold change of biofilm expression relative to planktonic expression ± standard error of the mean.

Figure S3: Ontological dendrogram displaying genome-wide expression of *Staphylococcus aureus* biofilms. Listed are homologous genes organized by KEGG ontological function hierarchies. USA300 locus tags are used throughout. The heatmaps depicts levels of preferential expression in biofilms (blue) or planktonic (red) populations at 5 h, 10 h, and 24 h. Values for colors were assigned based on RNA-seq fold-difference in expression between biofilm and planktonic cell population for each timepoint and each strain analyzed independently.

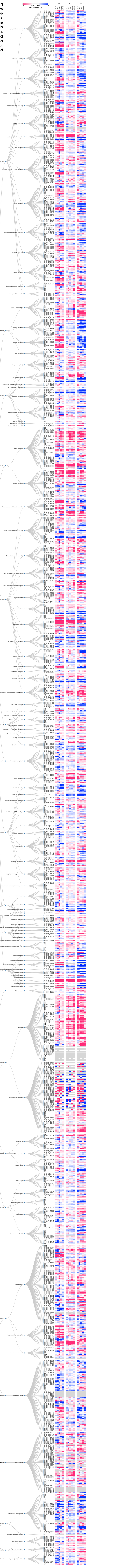

Supplement: Supplementary material 1 [file mgen-7-0598-s001.pdf]
